# A new relictual and highly troglomorphic species of Tomoceridae (Collembola) from a deep Croatian cave

**DOI:** 10.3897/zookeys.69.739

**Published:** 2010-10-18

**Authors:** Marko Lukić, Céline Houssin, Louis Deharveng

**Affiliations:** 1 Croatian Biospeleological Society, Demetrova 1, HR-10000 Zagreb, Croatia; 2 Muséum National d’Histoire Naturelle, UMR7205 CNRS/MNHN, CP50, 45 rue Buffon, 75005 Paris, France

**Keywords:** New taxon, *Tritomurus*, Croatia, cave hygropetric, troglomorphy

## Abstract

Tritomurus veles **sp. n.** (Tomoceridae) is described from a Croatian cave. It is characterized by troglomorphic features (absence of eyes, reduced pigmentation, slender claw, pointed tibiotarsal tenent hairs) that only compare, among Tomoceridae, to the microendemic species Tritomurus falcifer from the Pyrénées. Tritomurus veles also shares with Tritomurus falcifer the absence of macrochaetae on head, a presumably non-adaptive character that within Tomoceridae is unique to these two species. Both species have no known epigean relatives in their respective distribution areas and can be considered as relictual.

## Introduction

The family Tomoceridae includes 149 species in 16 genera, grouped in two subfamilies, Tomocerinae with 131 species and Lepidophorellinae with 18 species (Bellinger et al. 2010). Tomocerinae are distributed across the whole Holarctic region, extending locally as south as the mountains of Northern Sumatra. They are conspicuous by their large size and abundance in forest litter, but are also diversified and frequent in the caves of different regions of Europe, eastern Asia and North America, with about 30 troglobitic species. Many of these cave species have a reduced number of eyes and reduced pigment (Christiansen 1964). However, few species exhibit strong morphological adaptation to cave life. The most remarkable species in this respect is Tritomurus falcifer Cassagnau, 1958, which apparently is limited to a small karst of the central Pyrénées. In the present paper, we describe from a Croatian cave a second highly troglomorphic species, Tritomurus veles sp. n., already recorded as Tomoceridae gen. sp. in Lukić and Deharveng (2008).We also introduce several new morphological characters for the taxonomy of Tomoceridae, discuss the validity of the genus Tritomurus and comment the world distribution of reduced-eyed Tomoceridae.

## Materials and methods

The first specimen of Tritomurus veles sp. n. was collected in 2001 during a speleological exploration of the pit named Amfora jama conducted by the Croatian Natural History Museum, Speleological section PDS Velebit, Speleological club SAK Ekstrem and the Nature Park Biokovo (Lukić and Deharveng 2008). Type material was collected during a recent visit of Croatian Biospeleological Society to the same pit.

Specimens were mounted on slides in Marc-André II, and were studied with a Leica DMLB microscope. Photographs of Figs 1–3, 13 and 35 were taken with a Jenoptik ProgRes C10+ camera mounted on a Leica DMLB microscope. SEM micrographs were taken with a Cambridge 600 scanning electron microscope. SEM material was coated with gold and kept on stubs in MNHN collections.

## Abbreviations

Abd.abdominal segment, Ant.antennal segment, Th.thoracic segment, Tita.tibiotarsus.

The chaetotaxic formula of bothriotricha is given per half-tergite from Th.II to Abd.V.

According to Yosii (1967), the dens of Tomocerinae is subdivided in three parts: proximal, medial and distal. We use the “short and developed” dental spines formula, derived from Folsom (1913) and Ågren (1903); spines of the proximal part of the dens cannot be differentiated here into small and large, because individual variability in their size is too high. The “short formula” of dental spines is: total number of spines in the proximal part of the dens / (number of small spines), (number of large spines, underlined) of the medial part of the dens. The “developed formula” includes the successive numbers of large spines (underlined) and number of small spines (not underlined) of the medial part of the dens.

## Taxonomy

### Genus Tritomurus

Frauenfeld, 1854

#### Type species:

Tritomurus scutellatus Frauenfeld, 1854: p. 17

#### Note:

Tritomurus scutellatus species was collected in “Grotte bei Treffen”; Treffen is the German name of Trebnje in Slovenia, where several caves are recorded in the literature.

#### Tritomurus veles sp. n.

urn:lsid:zoobank.org:act:24AA93DE-023A-4957-9F33-B9D5116F1E02

Figs 1 [Fig F2] [Fig F3] [Fig F4] [Fig F5] [Fig F6] [Fig F7] [Fig F8] [Fig F9] [Fig F10] [Fig F11] [Fig F12] 35 

##### Type locality.

Croatia, Biokovo Mt., Sveti Jure: Amfora jama. Coordinates: 43°20'48.5"N, 17°02'48.0"E (WGS84), elevation 1620 m.

##### Type material.

Holotype male, 7 paratypes on slides (5 females, 2 with sexual plate not observable), 12 paratypes in 96% alcohol, 2 paratypes metalized for SEM, 17 July 2008, leg. M. Lukić; 1 paratype on slide (male), 14 July 2008, leg. B. Jalžić; 3 paratypes in 96% alcohol, 17 July 2008, leg. G. Rnjak; 4 paratypes in 96% alcohol, 03 November 2001, 16 July 2008, 18 July 2008, leg. J. Bedek. All material collected by hand.

##### Type material deposition.

Holotype (CLL 969), 3 paratypes on slide (CLL 785, 794) and 17 paratypes in 96% alcohol (CLL 244, 785, 791, 792, 793) deposited in the collection of Croatian Biospeleological Society, Zagreb, Croatia.

5 paratypes on slide, 2 paratypes in 96% alcohol, 2 paratypes metalized for SEM observation deposited in the collections of the Muséum National d’Histoire Naturelle de Paris.

##### Derivatio nominis.

Named after Veles—a Slavic god of earth, water and the underworld.

##### Description.

Body length 3.4 to 3.7 mm. Habitus slender, color pale grey in alcohol with scattered black pigment and white patches (Figs 1, 2). Region around the base of Ant.I without pigment (Fig. 3). Narrow white median line from Th.II to Abd.II. Primary granulation of integument fine and regular, mostly composed of hexagonal meshes (Figs 6, 25, 32); some areas with fusion of primary granules resulting in quadrangular or irregular meshes (Fig. 15). Eyes absent, ocular spot weak (Fig. 3) or absent.

**Figure:**
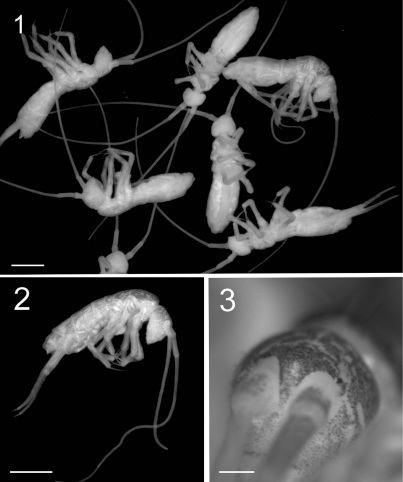
**Figures 1–3.** Tritomurus veles sp. n. (optical stereomicroscope). **1, 2** Habitus (scale 1 mm) **3** Head (scale 0.2 mm).

**Figure F2:**
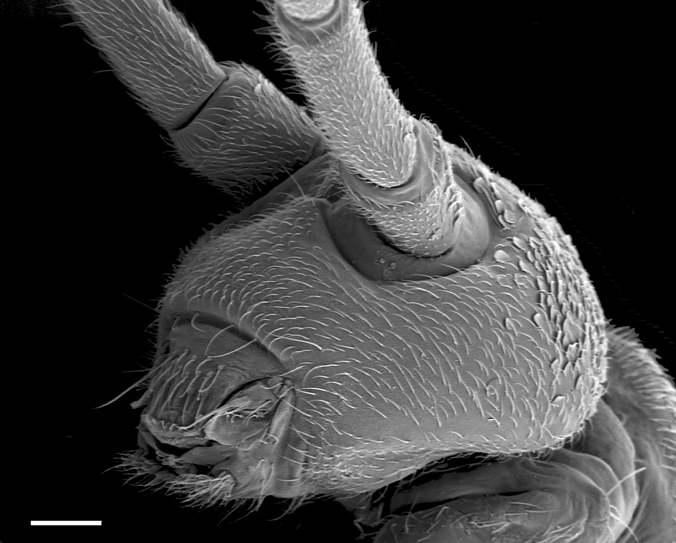
**Figure 4.** Tritomurus veles sp. n., head in lateral view (SEM, scale 100 μm).

**Figure F3:**
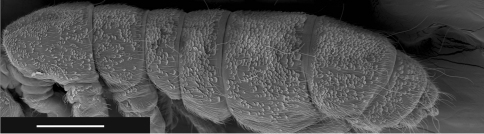
**Figure 5.** Tritomurus veles sp. n., body (SEM, scale 400 μm).

**Figure F4:**
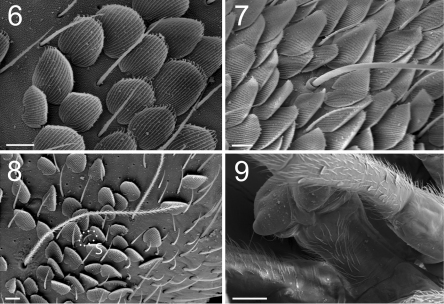
**Figures 6–9.** Tritomurus veles sp. n. (SEM). **6** Scales and ordinary mesochaetae on Th.II (scale 10 μm) **7** Scales, mesochaetae and macrochaeta on Abd.III (scale 10 µm) **8** Bothriotrichal area of Th.II, illustrating the presence of the four main chaetal types: bothriotricha, mesochaetae, S-chaetae (S) and scales (scale 10 µm) **9** Ventral tube in anterior view (scale 100 µm).

Body and appendages with four types of chaetae: ordinary chaetae, S-chaetae, bothriotricha and scales (Fig. 8). Other specialized chaetae present on labrum, maxillary palp, labial palp and mucro (see description of these organs below).

Ordinary chaetae numerous on body and appendages, slightly rugose at optical microscope magnification and longitudinally rugose-striate at higher SEM magnification, distally tapering, with well-marked thin sub-basal ring, differentiated as medium mesochaetae and long to very long macrochaetae; macrochaetae thin, acuminate, curved, not basally swollen, with socket ring markedly protruding above integument level (Fig. 7); mesochaetae basally swollen in specimens on slide but not in those examined with SEM; socket rings of mesochaetae strongly protruding on Ant.III-IV and dens (Figs 11–12, 31, 32), not protruding on tergites (Figs 6–8). Dense clothing of mesochaetae, particularly laterally (Figs 4, 5); macrochaetae few, frequently detached in microscopic preparations (Fig. 5); minute microchaetae present on anal valves and empodium.

**Figure F5:**
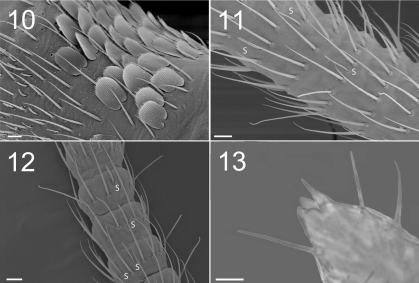
**Figures 10–13.** Tritomurus veles sp. n. (10–12, SEM; 13, optical microscope) **10** Lateral view of Ant.I, with scales, mesochaetae, and latero-distal short S-microchaetae at left (scale 10 µm) **11** Ant.III proximally (scale 10 μm) (S, S-chaetae) **12** Ant.IV (scale 10 μm) (S, S-chaetae) **13** Apical part of Ant.IV (scale 10 μm).

**Figure F6:**
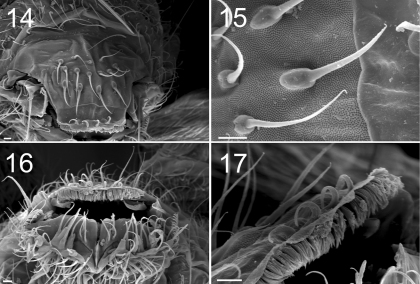
**Figures 14–17.** Tritomurus veles sp. n. (SEM) **14** Labrum (scale 10 μm) **15** Detail of labral chaetae with swollen socket (scale 10 µm) **16** Mouth (scale 10 μm) **17** ventro-distal brush of labrum (scale 10 μm).

**Figure F7:**
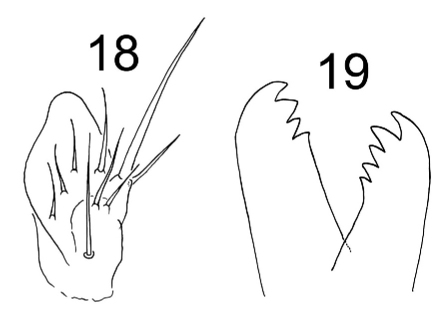
**Figures 18–19.** Tritomurus veles sp. n. **18** Outer maxillary lobe **19** Left and right mandibles.

S-chaetae smooth, subcylindrical, of variable length and thickness, not basally swollen, with socket border not or slightly protruding, located on antennae and tergites (Figs 8, 10–12); 1+1 very short (1/5 of surrounding mesochaetae), thin S-microchaetae ventro-laterally among antegenital mesochaetae (Fig. 34).

Bothriotricha thinner than macrochaetae, long, ciliated, present on Th.II, Th.III, Abd.III and Abd.IV, without marked protruding socket ring (Fig. 8).

Scales overlapping, evenly arranged (Figs 4, 5, 6–8, 10, 29–30), transparent, rounded, small, morphologically uniform except on furca, with 20–25 longitudinal ribs (Figs 6, 8); scale ribs parallel, entire, ending as ciliate processes beyond the distal edge of the scale (Figs 6, 10, 30). Scales present dorsally from head to Abd.VI (Figs 4, 5), dorsally on Ant.I–II (Fig. 4), on most parts of legs, dorsally on manubrium and dens (Figs 29, 30). Scales absent from ventral side of head (Fig. 4), Ant.III–IV, sternites, all except the hind tibiotarsus, and ventral tube (Fig. 9). Manubrial scales larger than those of body, rounded except a distal group of elongate scales (Figs 29, 30); dental scales fusiform, narrower and much smaller than manubrial scales (Fig. 30).

Antennae 1.5–1.8 times length of body (Figs 1, 2). Antennal segment ratio as I:II:III:IV = 1:1.79:15:4.58. Microchaetae at bases of Ant.I and II not differentiated. Ant.I and II with a dense clothing of mesochaetae, and S-chaetae of two main types: (i) dark, thin, straight, long (usually longer than surrounding mesochaetae), evenly distributed in large number among mesochaetae; (ii) hyaline, variously but moderatly thickened, shorter than surrounding mesochaetae, less evenly distributed (mostly ventrally and distally) (Fig. 10). Ant.III and IV annulated. Basal part of Ant.III (Fig. 11) with irregularly arranged chaetae, progressively organized in whorls for most of its length, as well as on Ant.IV. Each whorl composed of a single row of 17–28 chaetae, including ordinary mesochaetae and a smaller number of S-like chaetae, mostly long and thin, some shorter and thicker (Figs 11, 12). Serial pattern not evident in chaetal arrangement across successive whorls. Ant.III sense organ not clearly differentiated. Two protruding papillae and a spiniform process (pin chaeta) simple, without lateral process apically on Ant.IV (Fig. 13); subapical organite not seen.

Labral formula 4/5,5,4. Prelabral chaetae smooth, curved, thinner and longer than the labral ones (Fig. 14); labral chaetae strong, smooth, acuminate, their socket with thickened appearance, fused to chaetal basis (Figs 14, 15); apical edge of labrum with four strong, recurved hooks (Figs 14, 17); distally, a large area devoid of primary granules well delimited by a transversal line at half distance between the apical edge of labrum and the distal row of chaetae. Ventro-distally, labrum with a thick brush (Figs 16, 17). Labium with about 30 baso-median chaetae and 5 baso-lateral chaetae; labial palp with numerous proximal chaetae (about 25), its distal part not examined in detail (Fig. 16). Outer maxillary lobe with one basal chaeta, a trifurcate palp with strong apical chaeta and 4 sublobal hairs (Fig. 18). Maxilla head stout, complex, similar to that of other Tomocerinae, with a strong external tridentate claw and without distinct maxillary beard-like extension on lamella 5; other lamellae not analyzed in detail. Mandibles asymmetrical, the left one with 4 teeth, the right one with 5 teeth (6 specimens observed, Fig. 19). Ordinary mesochaetae numerous on the clypeus (Fig. 4). Hypopharynx well developed with ciliate processes.

Dorsal chaetotaxy dense and regular on head and tergites, consisting of small rounded scales, mesochaetae and a few macrochaetae, but no microchaeta (Fig. 5). Chaetal row along the posterior part of antennal basis field made of mesochaetae identical to those of the head. A line of numerous (more than 30) short equal mesochaetae regularly and closely arranged at the posterior edge of head. Clothing of mesochaetae on tergites denser where scales are absent, i.e. laterally and behind posterior row of macrochaetae, especially on Abd.IV and Abd.V (Fig. 5); mesochaetae more variable in size behind posterior macrochaetae than on the remaining of these tergites.

Macrochaetotaxy and bothriotrichal patterns of tergites illustrated in Fig. 20. Macrochaetae per half-tergite: none on head; 1 anterior and 2 posterior on Th.II; 2 posterior on Th.III; 2 posterior on Abd.I; 2 posterior on Abd.II (one lateral mesochaeta almost the length of the macrochaetae); 1 antero-median, 1–2 latero-median and 5 postero-lateral on Abd.III; 5–6 posterior, 1 antero-lateral and 5–6 lateral on Abd.IV; 4 posterior in two groups on Abd.V. Antero-median macrochaetae of Abd.III and postero-internal macrochaetae of Abd.IV and Abd.V very long, longer than Abd.V length; other macrochaetae shorter than Abd.V length. Long lateral mesochaetae present mostly on Th.III, Abd.I and Abd.II. S-chaetae thinner, shorter and much less numerous than mesochaetae, arrangement on tergites not clear. Bothriotrichal formula 0/2,1/0,0,1,2,0. Bothriotricha slightly shorter than long antero-median macrochaetae on Abd.III and than longest macrochaetae on Abd.IV; external bothriothrix about three-fourths as long as internal bothriothrix on Abd.IV. Macrochaetae without circlet of spine-like microchaetae near their bases. Scale arrangement and size not modified around macrochaetae sockets; 6–7 scales smaller in size ahead each bothriotrix of Abd. III-IV, more densely arranged than on the remaining of the tergites. Abdominal segment ratio as I:II:III:IV:V:VI = 1:1.37:2.53:2.26:1.16:0.68.

**Figure F8:**
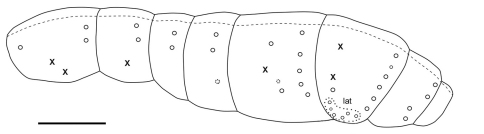
**Figure 20.** Tritomurus veles sp. n., macrochaetotaxic and bothriotrichal pattern; plain line circles, macrochaetae; dotted line circles, small macrochaetae or large mesochaetae; lat, lateral group of Abd.IV; X, bothriotricha) (scale 400 μm).

**Figure F9:**
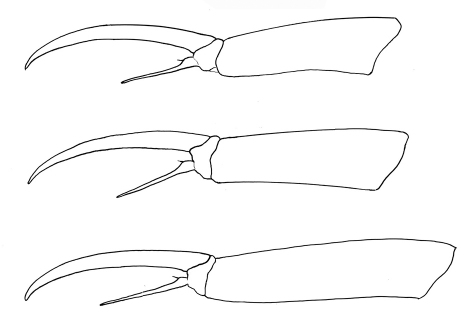
**Figure 21.** Tritomurus veles sp. n. Tibiotarsus and claw of legs I, II, III from upper to lower.

**Figure F10:**
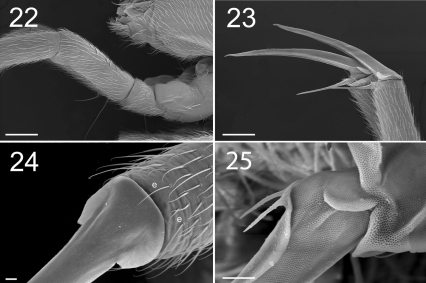
**Figures 22–25.** Tritomurus veles sp. n. (SEM). **22** Leg I, with ventro-basal macrochaetae of femur and ventral macrochaetae of trochanter (scale 100 μm); the second visible macrochaetae of femur belongs to other leg **23** Claws of legs I (scale 100 µm) **24** Claw of leg I, basal part in dorsal view (scale 10µm); e, thin distal tenent hairs **25** Bifurcate empodial appendage of leg II (scale 10 μm).

Claw very slender, without inner tooth (Fig. 23), devoid of internal ridging characteristic of other Tomocerinae. Basal wings of claw wide and short (12–15% of claw length), blunt, with truncated and denticulate apical edges (Fig. 24). Empodial appendage (emp) straight, thicker basally, parallel distally until the tip, about half the length of inner edge (i.e.) of claw (emp/i.e.= 0.45 for legs I and II, 0.55 for leg III) (Fig. 21), with long, thin internal tooth, this tooth often bifurcated, occasionally trifurcated (Figs 23, 25). Pretarsus with 1+1 minute microchaetae. Tibiotarsi I and II slightly shorter than outer edge (o.e.) of claw, tibiotarsus III slightly longer (o.e./Tita=1.14 for leg I, 1.07 for leg II, 0.91 for leg III) (Fig. 21). Distal whorl of tibiotarsi I, II and III with 11 or 12 acuminate chaetae, not distinct from other tibiotarsal chaetae, including dorsally 2–3,3,3 thinner, straight, acuminate chaetae  (Fig. 24), of which the most dorsal on each tibiotarsus probably corresponds to the tenent hair of other Tomocerinae. Clubbed tenent hairs absent. Tibiotarsi I, II and III with 0–1, 0–1 and 2–4 long, thin, dorsal macrochaetae, respectively; the proximal macrochaeta of tibiotarsus III nearly half the tibiotarsal length; other tibiotarsal chaetae as ordinary mesochaetae of variable length. Scales present dorsally from subcoxa to femur of legs II and III, on trochanter and femur of leg I (Figs 5, 9, 22), and dorso-basally on tibiotarsus III.

Each femur with very long, and thin ventro-basal macrochaeta, the two-thirds length of femur, inserted perpendicular to integument (Fig. 22); rest of femoral clothing mesochaetae. Each trochanter with one long, thin ventro-basal macrochaeta similar to that of femur (Fig. 22). Trochanteral-femoral organ not differentiated.

Ventral tube (Fig. 9) without scales, with about 60 mesochaetae, including 3 noticeably longer chaetae, on each latero-distal flap, anteriorly about 30–35+30–35 subequal, long mesochaetae, and posteriorly about 70 subequal mesochaetae.

Tenaculum with 1 or 2 chaetae and 4 teeth on each ramus. Manubrium dorsally with 2 longitudinal strips of mesochaetae and 2-3+2-3 erect macrochaetae, separated by a medial zone devoid of chaetae (Fig. 28); laterally with a row of chaetae on each side (Figs 28, 29); ventrally with large, rounded scales and a proximal group of ordinary chaetae (Fig. 29); ventro-distally, without chaetae but with a group of large, elongated scales (Figs 29, 30); ventro-distal sclerifications of manubrium with 1+1 internal triangular protrusions. Dens without outer basal spine-like chaetae or inner basal scale-like spine; ventro-basally with dense cover of small, narrow, fusiform scales (Fig. 30) turning to mesochaetae distally; externally with ordinary chaetae mixed with scales; dorsally, with dense clothing of subequal mesochaetae similar to those of manubrium; internally with basal rugose spine series (Figs 31, 32), the spines slightly inflated basally. Dental spines 4–5 in two irregular rows in the proximal part; 9–13 in one row in the distal part, variously arranged, usually asymmetrically (Fig. 26). Formula (and corresponding short formula) for 9 specimens:

5/1,1,1,1, 2,1,2,1 (short: 5/6, 4)

4/1,1,2,1,3,1 (short: 4/6, 3)

5/2,1,1,1,4,1 (short: 5/7, 3)

4/2,1,2,1,3,1 (short: 4/7, 3)

5/2,1,2,1,3,1 (short: 4/7, 3)

6/1,1,1,1,1,1,1,3,1 (short: 6/6, 5)

?/1,1,1,4,1,3,1 (short: ?/8, 4)

5/1,3,1,4,1,2,1 (short: 5/9, 4)

4/2,4,1,4,1 (short: 4/8, 4)

Mucro with two irregular internal lamellae without intermediate tooth (Fig. 27), covered of ordinary chaetae with elongated, protruding sockets (Fig. 33). Basal teeth unequal, small, without toothlet. Ratio mucro : dens : manubrium = 1:8.08:5.33

Antegenital chaetae numerous, all of the ordinary type, except for one minute S-chaeta on each side (Fig. 34). Female genital slit with 1+1 anterior microchaetae. Two microchaetae on each anal valve. Male genital plate rounded, with many mesochaetae.

**Figure F11:**
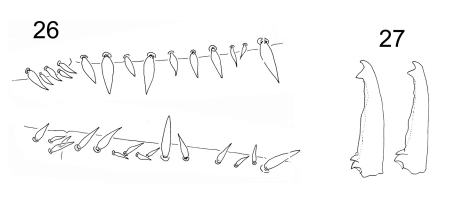
**Figures 26–27.** Tritomurus veles sp. n. **26** Dental spines formula in a female specimen: 4/2,4,1,4,1 (lower, right dens) and 5/1,1,1,1, 2,1,2,1 (upper, left dens) **27** Mucro in two different specimens.

**Figure F12:**
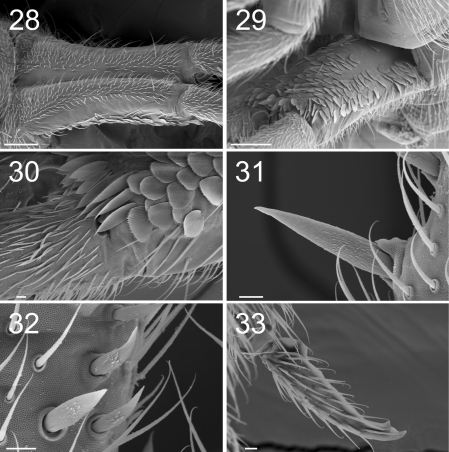
**Figures 28–33.** Tritomurus veles sp. n. (SEM). **28** Manubrium in dorsal view (scale 100 μm) **29** Manubrium in ventral view (scale 100 μm) **30** Manubrium ventro-distally and dens ventro-basally (scale 10 μm) **31, 32** Dental spines (scale 10 μm) **33** Mucro (scale 10 μm).

**Figure:**
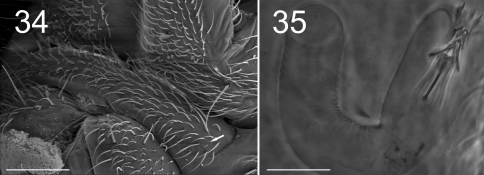
**Figures 34–35.** Tritomurus veles sp. n. (34, SEM; 35, optical microscope). **34** Sternite of Abd.V with genital plate (scale 100μm); arrow points to minute lateral S-microchaeta **35** Internal parasitic larva (Nematomorpha) (scale 20 µm).

## Discussion

### Ecology

Tritomurus veles sp. n. was collected from -170 to -430 meters in the deep pit named Amfora jama. All specimens were found far away from entrance, in total darkness on or adjacent to the thin water-film flowing on vertical walls (hygropetric habitat). They walked on the water film with legs widely spread; if the water current or water drops detached them from the wall, they floated downstream and obtained purchase in another place. While walking on the wall with thin water-film, only the legs were immersed in water while body was held above the water surface. This species was not observed entering or on the surface of the pools. Water temperature at a depth of -350 meters was 4.7°C and air temperatures were 5.1–5.4ºC.

The morphology and environment of the new species are similar to that of Tritomurus falcifer, which lives in the hygropetric habitat of caves on the Arbas massif of the French Pyrénées. Both species have the ventro-distal labral brush particularly well developed, apparently more than other epigean representatives of the family. This mouthpart modification recalls similar filtering structures observed in other species of the cave hygropetric (Moldovan et al. 2004; Sket 2004), and suggests special feeding habits. The guts of the collected specimens were filled with very fine clay-like material, without the mycelium or spores typical of epigean Tomocerinae. The new species probably ingests clay as do many other troglobitic Collembola (unpublished observations).

Large parasitic worms (larva of Nematomorpha), were visible inside the body of several specimens (Fig. 35).

Tritomurus veles sp. n. was found with the beetle Radziella styx Casale & Jalzic, 1989 (Coleoptera, Leiodidae), another obligate inhabitant of the cave hygropetric. It supplements the already remarkable endemic fauna of Amfora jama, which includes Dina sp. (Hirudinea), Zospeum sp. (Gastropoda), Protoneobisium biocovense (Müller, 1931)and Neobisium peruni Ćurčić, 1988 (Pseudoscorpiones), Speoplanes giganteus biocovensis Müller, 1934 (Leiodidae), Biokovoaphaenopsis radici Jalzic, 1993 (Coleoptera, Carabidae), Alpioniscus sp. (Oniscida), Oncopodura sp., Pseudosinella sp. and Verhoeffiella sp. (Collembola), and Biokoviella mauriesi Mršić, 1992 (Diplopoda).

### Relationships

Tritomurus veles sp. n. is strikingly similar at first sight to the rare cave species Tritomurus falcifer Cassagnau from Pyrénées by obvious troglomorphic traits: slender habitus, pigment reduction, anophthalmy, claw elongation, and reduction of tenent hairs to short, pointed chaetae. Such characters are assumed to be adaptive to cave life, and occur in most obligate subterranean species of Collembola (Thibaud and Deharveng 1994). But the two species also share several non-troglomorphic characters, including those listed as defining the genus Tritomurus by Bellinger et al. (2010): presence of scales on the body, absence of post-antennal organ and of eyes, trochanteral and femoral organs not differentiated, mucro elongate and setose with two basal teeth, the outer one devoid of toothlet, dens without large basal outer macrochaetae and without inner basal scale-like spine. Two other characters not considered as adaptive in the literature, the absence of macrochaetae on head and the absence of internal lobulations on claw, are also unique to Tritomurus falcifer and Tritomurus veles sp. n. among Tomocerinae.

Differences between Tritomurus veles sp. n. and Tritomurus falcifer include pigmentation (Tritomurus veles sp. n. has traces of pigment, Tritomurus falcifer is totally white), claw elongation (claw slightly more slender in Tritomurus veles sp. n.), claw structure (teeth absent on inner edge of claw in Tritomurus veles sp. n., present in Tritomurus falcifer), tooth on empodial appendage (present in Tritomurus veles sp. n., absent or inconspicuous in Tritomurus falcifer), and number of chaetae on tenaculum (Tritomurus falcifer has 5 chaetae, Tritomurus veles sp. n. has 1–2).

According to the most recent generic key for Tomoceridae (Bellinger et al. 2010), Tritomurus veles species is a member of Tritomurus. This genus is, however, poorly defined, as indicated by Cassagnau (1958), and the assignment of Tritomurus falcifer and Tritomurus veles sp. n. to Tritomurus is unsatisfying. Tritomurus scutellatus Frauenfeld, 1854 from Slovenian caves, the type species of the genus, differs from *falcifer* and *veles* sp. n. in significant characters, including the presence of cephalic macrochaetae and a much different claw structure (claw not elongate and with very large basal wings in Tritomurus scutellatus). The features that separate Tritomurus falcifer and Tritomurus veles sp. n. from Tritomurus scutellatus and from all other Tomocerinae may well justify placing them in a separate genus. The placement of these species in Tritomurus is therefore provisional, pending redescriptions of Tritomurus scutellatus and Tritomurus falcifer based on fresh material.

### Troglomorphy

All species of Tomocerinae living outside caves have 5+5 or 6+6 eyes. Those living in caves may have the complete 6+6 eye set for the family (e.g., Tomocerus problematicus Cassagnau, 1964 in the Pyrénées and Plutomurus unidentatus (Börner, 1901) in central and northern Europe), or a reduced number of eyes, but all species with reduced eye number are cave-restricted. Among reduced-eyed species, several also exhibit partial or total loss of pigment and have pointed tenent hairs, two characters often observed in subterranean Collembola. Claw elongation, considered another correlate to cave life in most troglomorphic Collembola (Christiansen 1961), is not observed in cave Tomoceridae, except Tritomurus falcifer and Tritomurus veles sp. n.

### Distribution

The species is only known from the type locality. A number of caves were explored during the five-year project 2002–2006 “Inventory and Mapping of the Subterranean and Spring Fauna of Biokovo Nature Park,” but Tritomurus veles sp. n. was not collected in any other cave, perhaps because hygropetric zones in most of these caves were practically inaccessible. Tomoceridae with reduced numbers of eyes (less than 4+4) belong to four genera: Lethemurus Yosii with 2 blind species from Japan and North America; Plutomurus Yosii with 9 species and 3 subspecies with 3+3 or fewer eyes, from Eastern Asia and North America; Tomolonus Mills with one 3+3-eyed species from North America; and Tritomurus with 3 blind species, including Tritomurus velves sp. n. European cave species of Plutomurus, the most diverse of these genera, have the full complement of 6+6 eyes except Plutomurus sorosi Kniss & Thibaud, 1999 from Georgia with 4–5 eyes per side. Thus, Tritomurus is the only genus of Tomoceridae with blind and fully troglomorphic species in the Western Palaearctic region. Both Tritomurus falcifer and Tritomurus veles sp. n. have extremely narrow distribution and no close relatives is known in their respective regional fauna which are relatively well sampled, as well as among Tomocerinae, suggesting a relictual status.

## Supplementary Material

XML Treatment for Tritomurus
